# The roles of testicular nuclear receptor 4 (TR4) in male fertility-priapism and sexual behavior defects in TR4 knockout mice

**DOI:** 10.1186/1477-7827-9-138

**Published:** 2011-10-13

**Authors:** Loretta L Collins, Yi-Fen Lee, Huei-Ju Ting, Wen-Jye Lin, Ning-Chun Liu, Charles K Meshul, Hideo Uno, Bo-Ying Bao, Yen-Ta Chen, Chawnshang Chang

**Affiliations:** 1 George Whipple Lab for Cancer Research, Departments of Pathology, Urology, Radiation Oncology, and The Cancer Center, University of Rochester Medical Center, Rochester, NY 14642, USA; 2 Research Services, V.A. Medical Center and Department of Behavioral Neuroscience, Oregon Health & Science University, Portland, OR 97239, USA; 3 Wisconsin Regional Primate Research Center, University of Wisconsin, Madison, WI 53708, USA; 4 Sex Hormone Research Center and School of Pharmacy, China Medical University, Taichung, Taiwan; 5 Department of Urology, Chang Gung University, Kaohsiung 833, Taiwan

**Keywords:** TR4, priapism, sexual behavior

## Abstract

**Background:**

Successful reproductive efforts require the establishment of a situation favorable for reproduction that requires integration of both behavior and internal physiological events. TR4 nuclear receptor is known to be involved in male fertility via controlling spermatogenesis, yet its roles in regulating other biological events related to reproduction have not been completely revealed.

**Methods:**

Male TR4 knockout (TR4-/-) and wild type mice were used for the sexual behavior and penile dysfunction studies. Mice were sacrificed for histological examination and corresponding genes profiles were analyzed by quantitative RT-PCR. Reporter gene assays were performed.

**Results:**

We describe an unexpected finding of priapism in TR4-/- mice. As a transcriptional factor, we demonstrated that TR4 transcriptionally modulates a key enzyme regulating penis erection and neuronal nitric oxide synthese NOS (nNOS). Thereby, elimination of TR4 results in nNOS reduction in both mRNA and protein levels, consequently may lead to erectile dysfunction. In addition, male TR4-/- mice display defects in sexual and social behavior, with increased fear or anxiety, as well as reduced mounting, intromission, and ejaculation. Reduction of ER alpha, ER beta, and oxytocin in the hypothalamus may contribute to defects in sexual behavior and stress response.

**Conclusions:**

Together, these results provide *in vivo *evidence of important TR4 roles in penile physiology, as well as in male sexual behavior. In conjunction with previous finding, TR4 represents a key factor that controls male fertility via regulating behavior and internal physiological events.

## Background

Members of the nuclear receptor superfamily are known to play important roles in differentiation, development, homeostasis, and metabolism, as well as in disease development and progression [1]. As a member of the nuclear receptor superfamily with several known regulatory targets [2], Testicular nuclear receptor 4 (TR4) may affect many signaling pathways and thus have a major impact on physiological functions [2]. Creation of mammalian gene knockout models has become a successful strategy to study the physiological roles of orphan receptors *in vivo *[3,4]. As a nuclear receptor with wide tissue distribution [5-10] and a relative mystery with respect to physiological function, TR4 was a good candidate for analysis using a knockout mouse model. A complex set of phenotypic abnormalities were found to exist in the TR4 knockout (TR4^-/-^) mouse, including significant growth retardation [11], defects in female reproductive function [12] and maternal behavior [11], impaired cerebella function [13,14], and reduced myelination [15]. In addition, recent studies found that TR4 might be a master regulator controlling glucose and lipid metabolism [16-18] as well as foam cell formation [19].

Our previous study revealed that TR4 expresses predominantly in the testis in a stage-dependent manner and deletion of TR4 in male mice reduced sperm production and disrupted spermatogenesis, thereby impaired male fertility [20]. Successful reproductive efforts require the establishment of a situation favorable for reproduction that requires integration of both behavior and internal physiological events. Following continue studies of TR4's roles in male reproduction, we describe an unexpected finding of priapism in some TR4^-/- ^mice. Penile priapism, or persistent erection, is characterized by trapped blood within the corpus cavernosa, which leads to reduced tissue oxygenation, increased blood viscosity, disruption of tissue elasticity, fibrosis, and finally irreversible failure to achive erection [21,22]. It is a significant urologic emergency that can lead to irreversible loss of erectile function if not promptly resolved. It has been linked to sickle cell disease as well as the use of vasoactive drugs, yet its molecular mechanism remains unclear. The etiology of this disorder is obscure. The cyclic nucleotide second messenger cGMP generated by activated guanylyl cyclase in penile smooth muscle cells regulates penile erection. These includes activation of guanylyl cyclase requires nitric oxide (NO), release in the penis upon sexual stimulation from neuronal and endothelial sources containing NO synthase (NOS), and respectively termed nNOS and eNOS. The crucial role of NOS in mediating erectile function was further supported by the finding that the mice lacking the gene for eNOS, nNOS, or both have a tendency for priapic activity [23]. To our surprise, in the mice with TR4 knockout [11], penile priapism was one of the most striking phenotypes observed.

In characterizing the fertility defects, in addition to partial penile priapism among TR4^-/- ^males, we also found that TR4^-/- ^mice displayed delayed sexual maturity, reduced sperm production [20], priapism, and abnormal sexual behavior, all of which contribute to significant reduction of TR4^-/- ^male fertility. Furthermore, the expressions of nNOS, enzyme involved in production of the erectile mediator NO, were reduced in penis tissue from TR4-knockout mice, where a transcriptional regulation of nNOS by TR4 was also presented.

## Methods

### Experimental animals and genotyping

TR4^-/- ^mice were produced as described [11], and housed in the vivarium facility of the University of Rochester Medical Center. The animals were provided a standard diet with constant access to food and water, and exposed to a 12-hour light/dark cycle. All experimental protocols were approved by the University Committee on Animal Resources and the office of Environmental Health and Safety prior to implementation.

Genotyping was carried out as described [11]. Briefly, genomic DNA was isolated from mouse tail samples and used as template for polymerase chain reaction (PCR). Primers for amplification of the wildtype and targeted alleles are TR4-107 (wildtype, forward): 5'-GGAGACACACTGCACATGTTCGAATAC-3', TR4-111 (wildtype, reverse): 5'-CACAGCTCATTTCTCTGCTCACTTACTC-3', Neo-3a (targeted allele, forward): 5'-GCAGCGCATCGCCTTCTATC-3', and TR4-34 (targeted allele, reverse): 5'-TGCAAGCATACTTCTTGTTCC-3'.

### Tissue preparation, histology, and immunostaining

Mice were anesthetized with an overdose of pentobarbital and perfused through the left ventricle with 20 ml saline (pH 7.3), followed by 20 ml of 4% paraformaldehyde or 10% neutral buffered formalin. Tissues were removed and post-fixed by submersion in 10% formalin. Alternatively, fresh tissues were fixed by direct submersion, in 10% neutral buffered formalin, prior to processing. Tissues were processed for embedding in paraffin using an RHS Tissue Processing System (Hacker Instruments & Industries), or processed manually. Penis tissues from 4.5-7.5 mo. old mice were cut in 5 μm sections, deparaffinized, and stained with Accustain Masson Trichrome (Sigma-Aldrich) reagents, according to the manufacturer's instructions. Additionally, penis sections were stained using a polyclonal antibody against nNOS (BD Transduction Laboratories) at a 1:200 dilution, a polyclonal antibody against S100 (DAKO) at a 1:500 dilution, or a polyclonal antibody against TR4 (Santa Cruz) at a dilution of 1:200, followed by the appropriate biotinylated secondary antibody (1:1000 dilution), VECTASTAIN *Elite *ABC reagent (Vector Laboratories) and DAB substrate (Vector Laboratories). Hematoxylin was used as a nuclear counterstain following immunostaining.

### RT-PCR and Western Blot analysis of gene expression

For RT-PCR analysis, total RNA was isolated from penis tissue using TRIzol^® ^Reagent (Invitrogen). First strand cDNA synthesis was achieved using the Superscript™II RNase H Reverse Transcriptase kit (Invitrogen).

Real time quantitative RT-PCR was carried out, using SYBR Green PCR MasterMix, on the iCycler iQ™ PCR cycler and detection system (Bio-Rad Laboratories). Analysis of data obtained and calculation of relative gene expression was performed using the 2^-ΔΔC^_T _method [24]. For each gene analyzed, the expression level of the TR4^+/+ ^was set at 1 and relative expression in TR4^-/- ^samples was calculated.

For Western blot analysis, total protein was isolated from penis tissue using TRIzol^® ^Reagent (Invitrogen). A total of 75 μg protein per sample was separated on either a 6% or a 10% SDS-PAGE gel. After transfer to PVDF membrane, the blots were probed with either a polyclonal antibody against nNOS (BD Transduction Laboratories) at a 1:1000 dilution, or an antibody against β-actin, followed by the appropriate alkaline phosphatase-conjugated secondary antibody at a 1:2000 dilution. Signals were detected using a chromogenic substrate.

### Transient transfection/reporter gene assay

The mouse nNOS promoter plasmid pEx2 [25] was provided by Dr. Ted Dawson (Johns Hopkins University). CV1 cells were grown in Dulbecco's Modified Eagle's Medium (DMEM) with 10% fetal bovine serum, at 37°C, and with 5% CO_2_. Cells were cultured in 24-well plates (Corning) for 24 h. Transient transfection was carried out as described previously [26]. The ß-gal activity in whole cell lysate was measured with a luminometer using a chemiluminescence-based detection system (Reaction Buffer, Galacto-Star, TROPIX) according to the manufacturer's protocol.

### Electrophoretic mobility shift assay (EMSA)

TR4 proteins were synthesized in rabbit reticulocyte lysate (Promega), according to the manufacturer's instructions. The oligonucleotide probe (nNOS-NHR, bp -192 to -211, 5'-CTGGTCAACCTTGACTTCCT-3') was end-labeled with [γ]32P in a T4 polynucleotide kinase reaction (New England Biolabs). The EMSA assay was performed as described previously [26].

### Continuous mating and male sexual behavior analysis

For the continuous mating study, 5 TR4^+/+ ^and 5 TR4^-/- ^males at 5 months of age were each paired with an adult TR4^+/+ ^female for 4 months. The number of litters produced and the number of pups per litter were recorded. For male sexual behavior analysis, ovariectomized female mice of strain ICR, at 8-9 weeks of age, were injected with 12 μg estradiol valerate one day before mating and with 500 μg progesterone 4-7 hours before mating. At the time of mating, one primed female was paired with a 7-8 month-old TR4^+/+ ^or TR4^-/- ^mouse for either 90 min or 6 h, beginning between 18:00-22:00. Mating behavior was videotaped and subsequently scored for latency to, and number of mounts, intromissions, and ejaculations [27,28]. After the 6 h taped mating trial, experimental pairs remained together until the following morning. Female mice were then visually examined for the presence of a vaginal plug.

### Statistics

For sexual behavior data, means and standard deviations for behavior latencies and mean number of behavior displays, with associated ranges, are shown. Extremely high variation in both latencies and numbers of specific behavioral displays was observed both within and between groups. Differences in numbers of mice of a particular genotype displaying a particular behavior were analyzed by one-tailed, independent sample *t*-tests, assuming equal variances, after coding for the presence (scored as 1) or absence (scored as 0) of the behavior.

## Results

### TR4^-/- ^males exhibit penile priapism

**Figure 1:**
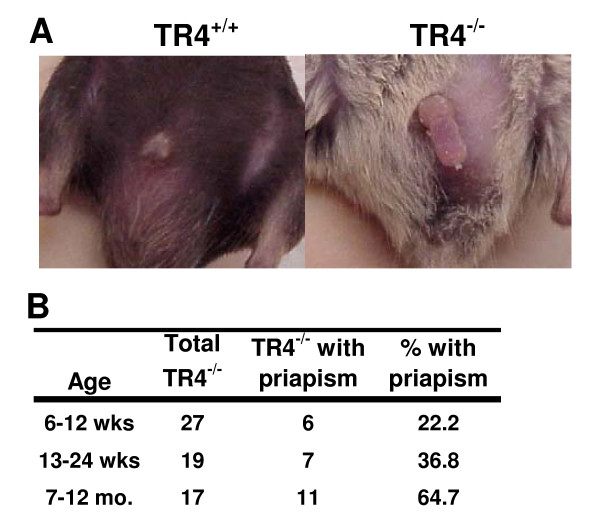
**TR4^-/- ^males display priapism. A**. TR4^+/+ ^male (left), and TR4^-/- ^male (right) showing priapism; both mice were 7 mo. old. **B**. Number and percentage of TR4^-/- ^mice showing priapism at various ages.

**Figure 2:**
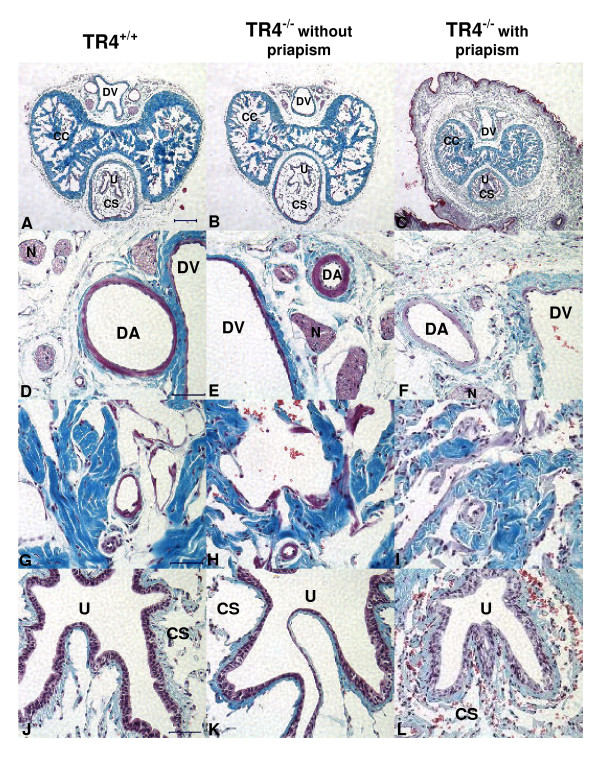
**Histological staining of penis sections from adult TR4^+/+^mice, and TR4^-/- ^mice both with and without priapism**. Penile cross sections are compared after Accustain Masson Trichrome (Sigma-Aldrich) staining. Each of panels G-I show a region of the corpus cavernosum from the sample indicated. DV, dorsal vein; DA, dorsal artery; CC, corpus cavernosum; CS, corpus spongiosum; N, nerve bundle; U, urethra. Scale is the same for all images in each row. For images A-C, scale bar = 250 μm; D-F, scale bar = 50 μm; G-I, scale bar = 50 μm; J-L, scale bar = 50 μm.

A physical defect observed among TR4^-/- ^male mice, which may affect sexual function, is partial priapism, or persistent, partial penile erection. This condition is observed in a substantial number, but not all of the TR4^-/- ^males, and increases in prevalence with age (Figure 1A and 1B). Once priapism was observed in a mouse, normal penile resheathing was not subsequently observed, suggesting persistence of priapism thereafter. As shown in Figure. 2, we observed more densely packed collagenous and elastic fibers in the corpus cavernosa from TR4^-/- ^mice with priapism, resulting in smaller cavernous sinuses, compared to TR4^+/+ ^or TR4^-/- ^without priapism (Figure 2C*vs *2A or 2B). Our previous studies on characterization of TR4^-/- ^mice found that heterozygote mice (TR4^+/-^) are indistinguishable with TR4^+/+ ^mice [11], and this remains true in this current study that TR4^+/-^, like TR4^+/+^, have no obvious defects. Therefore, we used TR4^+/+ ^as a control group in following studies. TR4 Comparing histological sections of penis tissue from a TR4^+/+ ^mouse (no priapism), a TR4^-/- ^mouse without priapism, and a TR4^-/- ^mouse with priapism (Figure 2D-L), we found more red blood cells within the corpus cavernosa (CC), and corpus spongiosum(CS) (H & I vs G) and dorsal vein (DV) (K & L vs J) in TR4^-/- ^tissue than in TR4^+/+ ^tissue.

### Regulation of nNOS transcriptional activation by TR4

**Figure 3:**
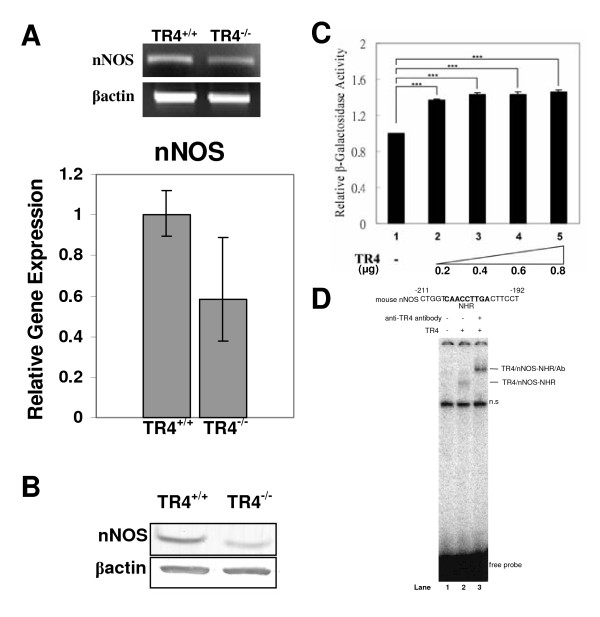
**Expression of nNOS is reduced in the TR4^-/- ^penis, and TR4 binds and activates the nNOS promoter**. **A**. Qualitative RT-PCR (upper panel) and real time RT-PCR quantitation (lower panel) of nNOS gene expression in the penis. Expression data were obtained, in triplicate, from each of two TR4^+/+ ^and two TR4^-/- ^mice. β-actin levels were determined as a control for template amount in PCR reactions. **B**. Western blot analysis of penis samples from TR4^+/+ ^and TR4^-/- ^mice (with priapism) demonstrates reduced nNOS protein expression in TR4^-/- ^tissue. Samples from each of 3 TR4^+/+ ^and 3 TR4^-/- ^mice were analyzed, and a representative blot is shown. **C**. Transactivation assay for TR4 regulation of nNOS expression. TR4 can activate the nNOS exon 2 promoter and control basal nNOS transcription. pEx2 (0.2 μg) and increasing amounts of TR4 were cotransfected into CV-1 cells. Promoter activity of each sample was normalized to the average activity of the pEx2 promoter when transfected alone. Results are the mean ± SEM from at least three experiments performed in duplicate. ***P < 0.01 d. By EMSA assay, TR4 is shown to bind to the nNOS-NHR probe (TR4/nNOS-NHR). The presence of TR4 in the complex was confirmed by supershift (TR4/nNOS-NHR/Ab) using a TR4-specific antibody. n.s., nonspecific binding.

To uncover TR4 roles in controlling penile activity, TR4 expression profiles were determined by the immunostaining of cross sectioned penis tissue. We observed TR4 expression in smooth muscle surrounding the penile vasculature (Additional File 1 Figure S1), a region where nNOS is also expressed [29]. A neuronal marker, S100, was used to confirm the dorsal nerve bundles in all sections. The penile erection requires NO released in the penis upon sexual stimulation. The synthetic enzyme for production of NO, a signaling molecule that is known to play a role in regulation of penile erection [30,31], was assayed in penis tissue. Through immunostaining for nNOS, we confirmed the colocalization of TR4 and nNOS in smooth muscle surrounding the penile vasculature, but also found significant variation in expression of nNOS, even within the same tissue (Additional File 1 Figure S1). To further quantify nNOS expression, quantitative real time RT-PCR with RNA from 7-month-old male TR4^-/- ^mice was used, and nNOS was found to be reduced by more than 65% relative to TR4^+/+ ^levels (Figure 3A). In addition to nNOS, we also found a significant reduction of mRNA expression of eNOS gene, another important NOS in regulating erection in TR4^-/-^(data not shown). Western blot analysis showed a clear reduction in the amount of nNOS protein present in TR4^-/- ^mouse penis tissue (Figure 3B). As a transcriptional factor, TR4 often regulates gene expression via binding to TR4 responsive element (TR4RE). Via sequence searching, a putative TR4RE was found in nNOS promoter. A reporter assay demonstrated that transfection of TR4 could enhance nNOS reporter gene expression (Figure 3C). The nNOS exon 2 contains a nuclear hormone receptor response element (NHR) through which the nuclear receptor SF-1 was found to bind and modulate nNOS transcription [25]. Using the EMSA assay, we demonstrated that TR4 protein can bind to the mouse nNOS NHR sequence (Figure 3D lane 2, TR4/nNOS-NHR), and this specific TR4-NHR complex can be super-shifted by addition of a TR4-specific antibody (lane 3, TR4/nNOS-NHR/Ab). In summary, we identified TR4 as a key modulator to control the penile function via a transcriptional regulation of nNOS expression, and loss of TR4 resulted in reduced nNOS expression that might contribute to penile priapism. TR4 represents a novel regulator that modulates nNOS gene expression to regulate penile function.

### Reduced fertility and aberrant sexual behavior in male TR4^-/- ^mice

Five adult TR4^+/+ ^males were mated with sexually mature female mice, producing a total of 22 litters. An average of 4.4 litters were produced per TR4^+/+ ^mouse in 4 months (Table 1). In contrast, only one of the 5 TR4^-/- ^males produced offspring, with a total of two litters born during the 4 month period (Table 1). Not only were significantly fewer TR4^-/- ^males able to produce offspring, the number of litters generated by the known fertile TR4^-/- ^male was approximately half that of the TR4^+/+ ^average (Table 1).

**Table 1:** Fertility rates of 5 month old TR4^+/+ ^and TR4^-/- ^males

TR4 genotype	Litters		Offspring	
	total	per male	total	per litter
+/+ (N = 5)	22	4.4	168	7.6
-/- (N = 5)	2	NA	13	6.5

Continuous mating with TR4^+/+ ^females for 4 month NA, not applicable since only one male produced offspring in the TR4^-/- ^group.

The initial male sexual behavior analysis that was carried out (Table 2 Trial 1) demonstrated defects in TR4^-/- ^sexual behavior, as well as social behavior. It was found that naïve TR4^-/- ^mice do not show defined sexual behavior within 90 minutes of the initial pairing with a primed female mouse. Out of 9 TR4^+/+ ^mice tested for sexual behavior, all 9 showed mounting behavior, 5 displayed intromission, and two achieved ejaculation, whereas none of the same behaviors were observed among the 11 TR4^-/- ^mice tested (Table 2). Interestingly, the TR4^-/- ^mice displayed fear or anxiety in the presence of a hormonally primed female, with a delay in showing interaction with the potential mate. In the most severe cases, the TR4^-/- ^male spent the entire test session actively avoiding his cage mate.

**Table 2:** Male sexual behavior

Behavior	TRIAL, 90 min		TRIAL, 6h	
	TR4^+/+^(N = 9)	TR4^-/- ^(N = 11)	TR4^+/+^(N = 4)	TR4^-/- ^(N = 6)
**Mounts**				
No. of mice	9/9^+^	0/11*	4/4^+^	5/6
Mean no. of mounts		0		33.8
Range	3-70		11-24	0-58
Latency to behavior ^++^			21.5 ± 20.0	136.8 ± 111.1*
**Intromissions**				
No. of mice	5/9	0/11*	4/4	2/6
Mean no. of mounts		0	14	7.2
Range	0-98		11-17	0-34
Latency to behavior ^++^			44.3 ± 32.1	89.8 ± 89.5
**Ejaculation**				
No. of mice	2/9	0/11*	4/4	1/6*
Latency to behavior ^++^			71.2 ± 58.6	91
**Plug Present**			4/4	0/6

^+^Number of mice showing behavior/number of mice tested; ^++ ^Mean ± standard deviation among animals showing behavior; Latencies are reported in minutes; *P < 0.05

Given extended pairing time, up to 6 h (Trial 2), TR4^-/- ^mice did begin to display sexual behavior. Again, proportionally fewer TR4^-/- ^than TR4^+/+ ^mice displayed each defined sexual behavior, with increased latencies to each behavior in TR4^-/- ^males (Table 2). After the videotaped 6 h pairing, male and female mice were left paired, and female mice were examined for vaginal plugs the following morning. Vaginal plugs were discovered in each of the female mice paired with TR4^+/+ ^males, whereas none of the females paired with TR4^-/- ^males had plugs (Table 2).

Expression levels of several genes known to affect behavior, particularly sexual behavior, were determined via semi-quantitative RT-PCR and quantitative real time PCR, using hypothalamic tissue from TR4^-/- ^and TR4^+/+ ^mice. RT-PCR results demonstrate that hypothalamic expression of estrogen receptor (ER) alpha (α), ER beta (β), and oxytocin is reduced in TR4^-/- ^mice (Additional File 2 Figure S2). No differences in levels of the androgen receptor (AR) or vasopressin (VP) were apparent. After quantitative real time PCR analysis, it was clear that the levels of ERα, ERβ, and oxytocin were reduced by approximately 50-60% in the hypothalamic region of TR4^-/- ^mice compared to TR4^+/+ ^(Additional File 2 Figure S2). More detailed mechanism dissection as to how TR4 regulates the expression of those genes involved in the sexual behavior and consequently affects reproductive function need further investigation.

## Discussion

In addition to the inability of the TR4^-/- ^mice with priapism to retract the penis back within the sheath (Figure 1A), histological evidence of priapism was observed (Figure 2). Trapped blood within the corpus cavernosum of the penis leads to pathology resulting from reduced tissue oxygenation and, without treatment, could result in permanent erectile dysfunction [21,22,32]. Compared with TR4^+/+ ^mice, both TR4^-/- ^animals with and without priapism have increased presence of red blood cells observed in penile cross section, with the largest amount present in sections of penis tissue from TR4^-/- ^mice with priapism (Figure 2L). Histology also revealed more densely packed tissue in the TR4^-/- ^mice with priapism, compared with TR4^+/+ ^mice and TR4^-/- ^mice without priapism, suggesting loss of elasticity of the normally flexible penile structures (e.g. corpus cavernosum, corpus spongiosum) (Figure 2C).

The neurotransmitter NO is a known mediator of erectile function that promotes vasodilation and the inflow of blood, resulting in penile tumescence [33]. nNOS is an enzyme responsible for production of NO in the penis [34], and expression of both nNOS mRNA and protein were reduced in the TR4^-/- ^mouse penis (Figure 3A and 3B). It was also found that TR4 is able to bind the NHR of exon 2 (Figure 3D), as well as upregulate nNOS gene expression in a reporter gene assay (Figure 3C). Immunostaining of penis tissue for TR4 and nNOS resulted in localization of both proteins in smooth muscle surrounding penile vasculature, further implicating TR4 in the regulation of vascular function via nNOS. As reported by Champion that NOS mutant mice: eNOS^-/- ^and eNOS^-/-^, nNOS^-/- ^displayed priapism where the expression of phosphodiesterase type 5 expression (PDE5A) was reduced, and transfection of eNOS normalized PDE5A expression and corrected priapic activity and there is less impact on loss of nNOS [23]. Therefore, in addition to nNOS, we also checked the expression of eNOS and PED5A in TR4^-/- ^penis, and found no significant difference from TR4^+/+ ^(data not shown). These results suggest that erectile function may be negatively affected by the loss of TR4 function in TR4^-/- ^mice, with or without priapism.

In addition to the possible loss of erectile function in TR4^-/- ^mice via reduced nNOS expression and/or resulting from the development of priapism, as well as the detrimental effects of loss of TR4 on male germ cell development [20], abnormal sexual behavior is a significant contributor to the reduced fertility of male TR4^-/- ^mice. For each sexual behavior demonstrated by TR4^-/- ^mice, the latency to behavioral display was increased compared to TR4^+/+ ^(Table 2). These results indicate that TR4^-/- ^mice still retain sexual motivation, yet take longer to become acclimated to the pairing situation and begin showing sexual behaviors. Furthermore, TR4^-/- ^males are less successful in achieving ejaculation. In fact, a large proportion of TR4^-/- ^males tested for 6 h did not even achieve intromission. From observation of the TR4^-/- ^mice during the mating trials, it became evident that they had difficulty in maintaining the appropriate mounting position through which intromission and ejaculation could be achieved.

TR4 ubiquitously expresses in almost all the tissues we examined, with high abundance in testis, and that leads to our first discovery of TR4 roles in male fertility via controlling spermatogenesis [20]. This current study further extends TR4's role as a master regulator in male fertility via controlling both sexual/social behavior and internal physiological events. This discovery points to the development of novel strategies for male fertility therapy that specifically target TR4. We have identified that polyunsaturated fatty acid could potentiate TR4 activity to control the lipid metabolism [19], and it will be of great interest to identify TR4 upstream modulators that can promote TR4 activity in male reproductive organs.

## Conclusions

Collectively, the data reported here describe severe reproductive malfunctions in TR4^-/- ^male mice, with significant effects stemming from abnormal sexual behavior, as TR4^-/- ^mice rarely achieve intromission or ejaculation. In TR4^-/- ^mice with priapism, erectile function may eventually be lost due to loss of collagenous tissue elasticity in the corpora cavernosa and corpus spongiosum, and account for the lack of intromission or ejaculation. In TR4^-/- ^males with or without priapism, disruptions of signaling pathways involving nNOS (Figures 3 and Additional File 1 Figure S1) may also affect erectile function. These *in vivo *data demonstrate that TR4 plays an important part in various aspects of reproduction and behavior, and future studies to more finely dissect the roles of TR4 in penile physiology and in brain/behavioral function may lead to identification of additional TR4 target genes, greater knowledge of the involvement of the receptor in various signaling pathways, or the discovery of new physiological ligand for TR4.

## Abbreviations

TR4: testicular orphan nuclear receptor 4; NOS: nitric oxide synthase; RT-PCR: reverse transcription-polymerase chain reaction; NHR: nuclear hormone receptor response element.

## Competing interests

The authors declare that they have no competing interests.

## Authors' contributions

LC, YFL carried out the TR4^-/- ^mice sexual behavior experiments and characterized penile priapsim phenotype and wrote the manuscript. HJT and BYB carried out eNOS and nNOS expression in gene and protein levels, WJL and YTC participated in sexual behavior experiments. NCL carried out nNOS reporter gene assay. CKM, HU and CC participated in its design and coordination and helped to draft the manuscript. All authors read and approved the final manuscript.

## Supplementary Material

Additional file 1 **Supplemental Figure S1**. Localization of both TR4 and nNOS to vascular smooth muscle in the mouse penis. Penis sections from adult TR4^+/+ ^and TR4^-/- ^(both with and without priapism) mice were stained for neuronal nitric oxide synthase (nNOS), the neuronal marker S100, or TR4 protein, as indicated. Immunoreactivity for both nNOS (upper panels) and TR4 (lower panels) was found in the smooth muscle surrounding veins (DV, dorsal vein) and arteries (DA, dorsal artery), as well as venules (V) and arterioles (A), of the penis. TR4 immunoreactivity is shown in an arteriole of the corpus cavernosum of a TR4^+/+ ^mouse, whereas no immunoreactivity was observed in the same structures from TR4^-/- ^mice. S100 immunoreactivity was observed in dorsal nerve bundles in all sections probed for the protein. CC, corpus cavernosum; CS, corpus spongiosum. Click here for file

Additional file 2 **Supplemental Figure S2**. RT-PCR and real time PCR analysis of sexual behavior/function-related gene expressions in the hypothalamus. A. Qualitative RT-PCR analysis of AR, ERα, ERβ, VP, and OT mRNA expression. β-actin levels were determined as a control for template amount in PCR reactions. B. Real time PCR quantitation of those genes expressing in the hypothalami of TR4^+/+ ^and TR4^-/- ^mice. Relative gene expression levels are shown. For both RT-PCR and real-time RT-PCR analyses, triplicate data from each of three TR4^+/+ ^and three TR4^-/- ^mice were obtained. Click here for file
