# Supplementary material for: The roles of testicular nuclear receptor 4 (TR4) in male fertility-priapism and sexual behavior defects in TR4 knockout mice
Source: Reprod Biol Endocrinol. 2011 Oct 13;9:138. doi: 10.1186/1477-7827-9-138 (PMC3212810; doi:10.1186/1477-7827-9-138)

Supplemental Figure S1

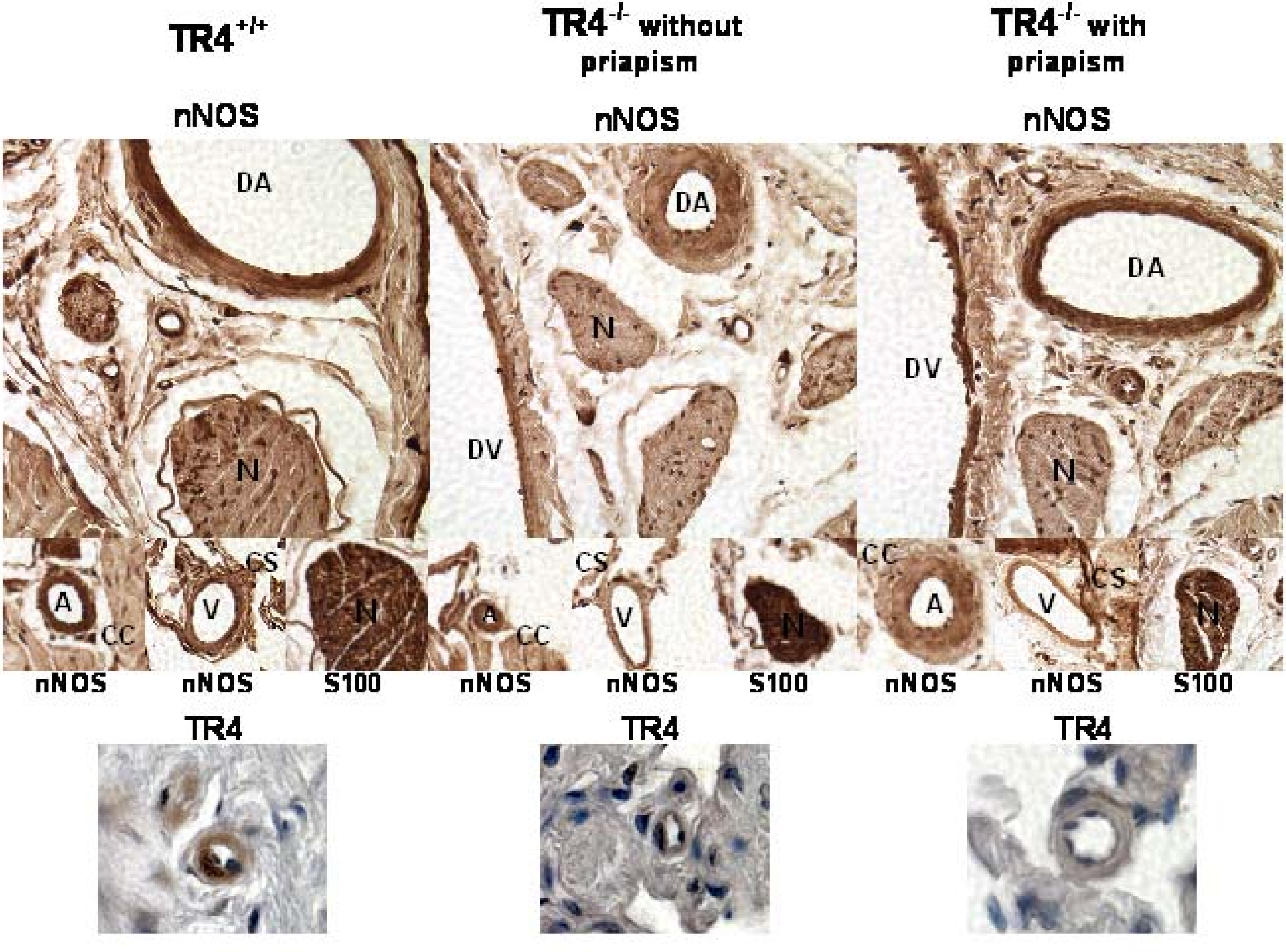

Supplement: Additional file 1 — Supplemental Figure S1. Localization of both TR4 and nNOS to vascular smooth muscle in the mouse penis. Penis sections from adult TR4+/+ and TR4-/- (both with and without priapism) mice were stained for neuronal nitric oxide synthase (nNOS), the neuronal marker S100, or TR4 protein, as indicated. Immunoreactivity for both nNOS (upper panels) and TR4 (lower panels) was found in the smooth muscle surrounding veins (DV, dorsal vein) and arteries (DA, dorsal artery), as well as venules (V) and arterioles (A), of the penis. TR4 immunoreactivity is shown in an arteriole of the corpus cavernosum of a TR4+/+ mouse, whereas no immunoreactivity was observed in the same structures from TR4-/- mice. S100 immunoreactivity was observed in dorsal nerve bundles in all sections probed for the protein. CC, corpus cavernosum; CS, corpus spongiosum. [file 1477-7827-9-138-S1.PDF]
