# Supplementary figures and images for: The roles of testicular nuclear receptor 4 (TR4) in male fertility-priapism and sexual behavior defects in TR4 knockout mice
Source: Reprod Biol Endocrinol. 2011 Oct 13;9:138. doi: 10.1186/1477-7827-9-138 (PMC3212810; doi:10.1186/1477-7827-9-138)

# Supplemental Figure S2

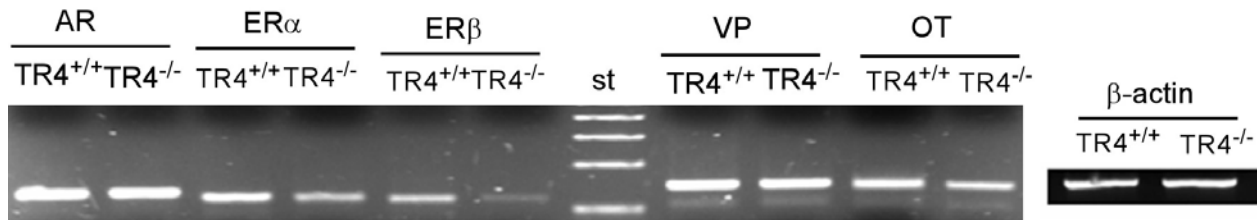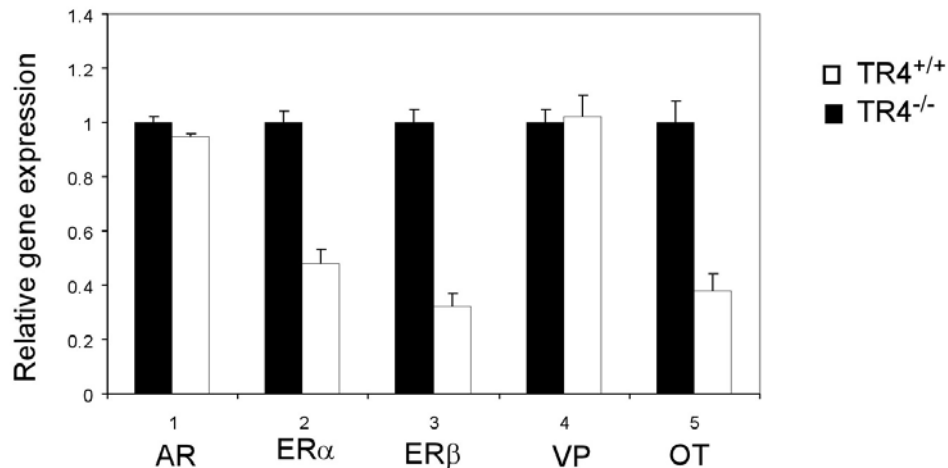

Supplement: Additional file 2 — Supplemental Figure S2. RT-PCR and real time PCR analysis of sexual behavior/function-related gene expressions in the hypothalamus. A. Qualitative RT-PCR analysis of AR, ERα, ERβ, VP, and OT mRNA expression. β-actin levels were determined as a control for template amount in PCR reactions. B. Real time PCR quantitation of those genes expressing in the hypothalami of TR4+/+ and TR4-/- mice. Relative gene expression levels are shown. For both RT-PCR and real-time RT-PCR analyses, triplicate data from each of three TR4+/+ and three TR4-/- mice were obtained. [file 1477-7827-9-138-S2.PDF]
